# Editorial Notes

**Published:** 1889-02

**Authors:** 


### HOMŒOPATHIC PHYSICIAN,

A MONTHLY JOURNAL OF

HOMŒOPATHIC MATERIA MEDICA AND CLINICAL MEDICINE.

"If our school ever give up the strict inductive method of Hahnemann, we are lost, and deserve only to be mentioned as a caricature in the history of medicine."—CONSTANTINE HERING.

Vol. IX. FEBRUARY, 1889. No. 2.

### EDITORIAL NOTES.

THE USE OF BOOKS AT THE BEDSIDE.—For one to place his whole confidence for the curing of the sick in his ability to properly use books at the bedside is very much like death-bed repentance; it may be done, but it is very unsafe to trust to it.

The discussion of the proper method of studying and using the materia medica is a very useful one, and a continuation of the subject, if conducted in a proper spirit, cannot fail to be of great advantage to us all. Some tell us that it is not necessary to study our materia medica; that it is no use to try to commit these long lists of symptoms to memory. In place of this study we are to carry with us to the sick-room such books as we need, and there to study out our case. Others, again, advise us to try to learn and to remember the leading symptoms of our drugs, and from such knowledge to select our remedies as we need them without any reference to books. We respectfully submit that both of these advisers are right to a certain degree. We should carefully study our drugs until we know them; each drug has a certain character, an individuality; this we should know. We cannot remember all the symptoms of each drug, but we can remember the peculiar traits (so to speak) of each, and its general range of action. When, in an emergency, we desire to know more we can consult our books. When we carry books with us to the sick-room, they are intended to assist our memory, not to replace it; we merely use them to refresh our memory.

When visiting patients, it has always been our habit to carry some book or other to assist us in case of need, yet the cases where any real assistance has been derived from such use of books have been very few indeed. The sick-room is no place for proper study; all the surroundings of a sick-bed tend to distract and disturb one's thoughts. Our rule has always been to take down the case, and if no remedy was clearly and safely indicated to give the patient sac. lac. Then the notes are taken home, and the case is quietly and carefully studied out in the office, where books are plenty and no one present to disturb. If the case be an urgent one, the next visit can be made as soon as the remedy is chosen. Any excuse may be made for this second visit, and the time spent at the office in searching for the proper remedy *will not be time lost.* This course can be pursued even in the most urgent cases; yes, even in moribund cases. We quote a case from Dr. Dunham's practice to show the timid physician that he has, even in the most urgent cases, plenty of time to study out his remedy, and that when the true simillimum is found it will act so promptly as to quickly regain all time spent in studying out the case.

The case we refer to was quoted in our January issue by Dr. Wells (see page 9). This patient, a child, had been sick at least twelve hours when first seen by Dr. Dunham. At the end of this twelve hours the child was in a condition which Dr. Dunham considered "exceedingly ill," and, as the doctor acknowledges, about ten more precious hours were lost, yea, more than lost, by two bad prescriptions, and yet this moribund child rallied in two hours after being given the true homœopathic simillimum! Should not such experience as this teach us to make haste very, very slowly? Does it not tell us that no length of time properly spent in seeking the true homœopathic remedy is lost time? Let us suppose that Dr. Dunham had, in this case, spent these ten hours in studying out his case rather than in trying experiments with two prescriptions, would not the patient have been in better condition to be promptly acted upon by the simillimum?

One of the best homœopathic physicians America has ever produced was recently discharged by a lady patient because he consulted a book at her bedside. She said she could not have confidence in any doctor who had to consult a book when prescribing for her, yet this doctor had done her more good in a few weeks than her previous old-school doctors had done in several years.

We would sum up our advice upon this subject in a few words. First, learn all you can of the true indications for each remedy; especially learn to discriminate between related drugs.

And this can be better and more easily done by studying cases than by studying the materia medica drug by drug, for in this way we get an idea of drugs in their relation to diseased conditions.

Secondly, don't be afraid to give your patients sac. lac., nor to take time—to take all the time needed for the thorough studying out of your cases. This, once done, you will find the rest of the case very easy to treat.

Thirdly, take with you in your daily visits a repertory or a materia medica, as preferred, to help you in cases of urgent need. But never neglect the study of the materia medica nor believe you can do without this study if you carry books with you.

---

TELLING THE WHOLE TRUTH.—In a recent editorial we alluded to the teaching given students at the so-called Hahnemann College of this city in the materia medica and in the study of the *Organon*. We remarked that the students were certainly fed on very light diet in these branches. The editors of the *Medical Institute of the Hahnemann Medical College* seem to be very much hurt at this reflection upon their college. In their December, 1888, issue, we read this stinging retort:

> "While we defer to any facilities that the 'so-called' HOMŒOPATHIC PHYSICIAN may have for judging light diet, we are nevertheless sure that the diet furnished by Dr. Mohr in his lectures on Materia Medica and Therapeutics, and Dr. Morgan in his lectures on the *Organon*, is sufficiently strong to tax to the utmost the digestive functions of *all* who listen to those persons and *many* who ought to listen to them.
>
> "In Hahnemann College, which is not only '*so-called*,' but so *characterized*, every lecturer on the principles of Homœopathy makes a direct effort to implant those principles in the minds of his pupils, and to imbue them with the truth of the science they are studying."

In reply to this, we would say that our knowledge of the light diet given students at this so-called homœopathic college is derived from men who have studied there in the past or who are now studying there. Moreover, that these students tell us the lectures on materia medica are generally read to the class from Dr. Wood's text-book; that the lecturer will spend almost all of his lecture hour thus quoting to them the allopathic uses of the various drugs, and, when his time is nearly all spent, he will briefly and very hurriedly allude to a few homœopathic uses. This lecturer recently told his class that any man who called the hypodermic syringe a "squirt gun" (as the late Dr. Lippe always called it) was either a bigot or a fool, or he had no practice. So much for the light diet in materia medica.

As to the lectures on the *Organon*, there are none, at least, so we are told. The learned lecturer begins his course at the commencement of the session at old Father Hippocrates, and wends his way so slowly down the centuries that at the end of the session he will be one or two centuries away from Hahnemann's time. We are told this lecturer has never been known to reach Hahnemann. We have also been informed that the class often ask him when they are to hear some news of Hahnemann, and are always put off for a more convenient season. So much for the diet in the study of the *Organon*. Yet, in spite of these complaints we are told "every lecturer on the principles of Homœopathy makes a direct effort to implant those principles!" Tell the truth, the whole truth!

